# Hybrid Sequencing Resolved Inverted Terminal Repeats in the Genome of Megavirus Baoshan

**DOI:** 10.3389/fmicb.2022.831659

**Published:** 2022-05-10

**Authors:** Yucheng Xia, Huanyu Cheng, Jiang Zhong

**Affiliations:** Shanghai Public Health Clinical Center, State Key Laboratory of Genetic Engineering, Department of Microbiology and Immunology, School of Life Sciences, Fudan University, Shanghai, China

**Keywords:** mimivirus, megavirus, genomics, inverted terminal repeat, Nanopore sequencing

## Abstract

Mimivirus is a group of amoeba-infecting DNA viruses with linear double-strand genome. It is found to be ubiquitous in nature worldwide. Here, we reported the complete genome of a new member of Mimivirus lineage C isolated from a fresh water pond in Shanghai, China. Its 1,224,839-bp genome encoded 1,062 predicted ORFs. Combining the results of Nanopore, Illumina, and Sanger sequencing technologies, two identical 23,919 bp inverted terminal repeats (ITRs) were identified at both extremities of the viral linear genome, one of which was missing in the draft assembly based on Illumina data only. The discovery of ITRs of Mimivirus provided a new insight into Mimivirus genome structure.

## Introduction

The first report of amoeba giant virus, Acanthamoeba polyphaga Mimivirus (APMV), changed our perception and understanding about virus greatly (La Scola et al., 2003; Raoult et al., 2004; Suhre, 2005). Following APMV, new classes of giant viruses have been identified in amoeba and other organisms, such as Pandoravirus celtis (Legendre et al., 2019), Bodo saline virus (BsV) (Deeg et al., 2018), Yasminevirus (Bajrai et al., 2019), Tupanvirus (Abrahão et al., 2018), Medusavirus (Yoshikawa et al., 2019), etc. These viruses have extraordinarily large virions size, as well as large and complex genomes harboring not only hundreds of orphan open reading frames (ORFans), but also many proteins barely seen in other viruses, like translation-related proteins, repeat-containing proteins, etc. The amoeba giant viruses were classified into the Nucleocytoplasmic Large DNA Viruses group (NCLDV) together with *Poxviridae*, *Asfarviridae*, *Phycodnaviridae*, *Ascoviridae*, and *Iridoviridae* (Iyer et al., 2001; Yutin et al., 2009).

Members of *Mimiviridae* can be divided into several lineages, including lineage A (represented by APMV, La Scola et al., 2003), B (Acanthamoeba polyphaga moumouvirus, Yoosuf et al., 2012), C (Megavirus chiliensis, Arslan et al., 2011), and Tupanvirus (Abrahão et al., 2018), based on the phylogenetic analysis of conserved genes, like major capsid protein, family B DNA polymerase, D5-like primase-helicase, etc. New lineage has been proposed due to the isolation of new viruses (Takahashi et al., 2021). Using different gene or gene set may result in slightly different topology in the phylogenic tree, but the lineage classification remains unchanged (Arslan et al., 2011; Yoosuf et al., 2012; Assis et al., 2017; Abrahão et al., 2018; Takahashi et al., 2021). Mimivirus had a large and complex genome with a relatively conserved central region and highly diversified terminal areas (Arslan et al., 2011; Yoosuf et al., 2012). It was proposed that genome duplication played an important role in shaping the genome of mimivirus since one-third of mimivirus genes had at least one paralog (Suhre, 2005).

Inverted terminal repeat (ITR) is a DNA structure found at the end of some linear replicons and essential for the stability and replication of the replicon (Blackburn, 1991). Long ITRs were found in some NCLDVs with linear genome, such as poxvirus (Tulman et al., 2006, 2004; Mitsuhashi et al., 2014), ASFV (Meireles and Costa, 1994), and chlorovirus (Strasser et al., 1991; Quispe et al., 2017). These ITRs of NCLDVs were AT-rich and closely resembled ITRs of *Escherichia coli* prophage N15, *Borrelia burgdorferi* linear chromosome and linear plasmids (Hinnebusch and Barbour, 1991; Hinnebusch and Tilly, 1993; Volff and Altenbuchner, 2000). Raoult et al. (2004) identified inverted repeats of about 900 bp in length near the ends of APMV linear genome and suggested that Mimivirus genome might adopt a circular topology for replication using ITRs.

In this study, the complete genome of Megavirus baoshan (Me. baoshan), isolated from a fresh water sample of a crawfish farm in Shanghai, China, was resolved. A total of 1,062 protein-coding genes and nine tRNAs were predicted. Two identical 23,919 bp-long ITRs were identified in the terminal regions of the linear genome. This may provide new insights into the structure and function of mimivirus genome.

## Materials and Methods

### Virus Isolation

A water sample was collected from a fresh water pond of a crawfish farm, in Baoshan district, Shanghai, China. The sample was first filtered through a filter paper to remove mud, and then filtered through a 0.22-μm filter membrane (Merck Millipore, Darmstadt, Germany). The filter membrane was washed with 5 ml Page’s amoeba saline (PAS) (Abrahao et al., 2016) containing 50 mg/L thiabendazole, 10 mg/L vancomycin, 20 mg/L ciprofloxacin, and 20 mg/L rifampicin for 1–2 h at room temperature, and the elution was collected.

*Acanthamoeba castellanii* str. Neff (ATCC30010) were cultivated in Peptone-yeast extract with glucose medium (PYG) (Abrahao et al., 2016) on a 12-well culture plate at 27°C for 2 days and then the medium was replaced with PAS containing the antibiotic cocktail.

The elution of filter membrane was added to amoeba in the wells. After 3 days, most of the vegetative form of amoeba became round and floating, implying the existence of amoeba virus. The protocols of Mimivirus isolation and titration as well as recipes of solutions were adopted from literatures (Abrahao et al., 2016).

### Virus Cloning and DNA Preparation

The cloning of Me. baoshan was performed using an infected-cell dilution method as used for Mollivirus and Tokyovirus (Legendre et al., 2015; Takemura, 2016).

Genomic DNA of the cloned strain of Me. baoshan was prepared from the supernatant of infected *A. castellanii* using PureLinkTM Genomic DNA Mini Kit (Invitrogen, Carlsbad, CA, United States) following manufacturer’s protocol. The protocol of DNA extraction from Gram-negative bacterial cells was used. Viral DNA was quantified with a NanodropTM 2000/2000c spectrophotometer (Thermo Fisher Scientific, Carlsbad, CA, United States), as well as by fluorometric quantitation with a Qubit 3.0^®^ Fluorometer (Thermo Fisher Scientific).

### Viral Genome Sequencing and Assembly

High-quality genomic DNA (2 μg) was used for DNA sequencing, which was carried out by the BioMarker Technologies Inc. (Beijing, China). For Illumina sequencing, viral DNA was fragmented into 400–500 bp using Covaris M220 ultrasonic instrument. The DNA library was prepared using TruSeqTM DNA Sample Prep Kit (Illumina, San Diego, CA, United States) and sequenced on an Illumina HiSeq Xten with paired-end 250 bp sequencing. For Nanopore sequencing, it was performed following the manufacturer’s protocol (Oxford Nanopore Technologies, Oxford, United Kingdom).

Illumina and Nanopore reads were processed with fastp v0.21.0 (Chen et al., 2018). Filtered sequence data of high quality (Phred quality greater than Q15, adapter sequence removed) from both Nanopore and Illumina sequencing were uploaded to the NCBI of the SRA as ID PRJNA778649. The filtered Illumina Hiseq paired-end reads were assembled *de novo* using SOAPdenovo v2.04 (Luo et al., 2012) and SPAdes v3.15.2 (Bankevich et al., 2012) with K-mer 67, and corrected using GapCloser v1.12 (Kosugi et al., 2015) to obtain the draft assembly (GeneBank: MH046811.1-MH046830.1). The filtered Nanopore reads were assembled *de novo* using the Canu v1.5 (Koren et al., 2017), and the assembled contigs were then corrected by Pilon v1.23 (Walker et al., 2014) using the filtered Illumina Hiseq paired-end reads at high stringency to obtain the preliminary hybrid assembly.

### Sequence Correction

The alignments of Illumina or Nanopore reads were done by BWA v0.7.17-r1188 (Li and Durbin, 2009). Read sort and coverage of each base were done by Samtools v1.10 (Li et al., 2009). The visual exploration of high throughput data was conducted with QualiMap v2.2.1 (Okonechnikov et al., 2016) and Integrative Genomics Viewer (IGV) v2.11.3 (Thorvaldsdóttir et al., 2013). Nanopore long reads were extracted by FastQ_screen v0.12.1 (Wingett and Andrews, 2018).

Two long regions with high sequence similarity greater than 98% were found in the left and right terminals of the preliminary assembly. They were compared using BLAST+ (Camacho et al., 2009) using the mode of two-sequence alignment and the selection of program optimized for high sequence similarity, and the results were exported and visualized by Notepad++ to identify single nucleotide variants (SNVs) between them.

Sanger sequencing was used to verify the SNVs in the terminal homologous regions. Potential PCR primers were evaluated using the Primer-BLAST on NCBI (Ye et al., 2012), and SnapGene^1^ was used to maximize the probability of successful amplification. Amplification reactions were performed using 2x Hieff^®^ Canace PCR Master Mix (Yeasen, Shanghai, China) in a GeneAmp 2400 thermal cycler (Perkin-Elmer-Cetus, Norwalk, CT, United States), and amplification products were gel-purified using HiPure Gel Pure DNA Mini Kit (Magen, Guangzhou, China) and T/A-cloned using 5min TA/Blunt-Zero Cloning Kit (Vazyme, Nanjing, China). Multiple recombinants were subjected to Sanger sequencing (GeneWiz Technologies Inc., Suzhou, China), and the sequencing results were compared to identify any potential nucleotide variations. The results of Sanger sequencing were remapped onto the preliminary assembly of Me. baoshan using SnapGene to correct errors, and the final complete assembly was obtained (GeneBank: MH046811.2).

### Genomic Annotation

Protein coding sequences (CDSs) were identified using the GeneMarkS v4.28 (Besemer et al., 2001) and Prodigal v2.6.1 (Hyatt et al., 2010). Transfer RNAs were found using tRNAscan-SE v2.0 (Lowe and Chan, 2016) with the general tRNA model. The functional assignment of predicted genes was performed using a combination of BLASTP searching against the database of non-redundant protein sequence (NR) and clusters of orthologous groups of proteins (COG) (Tatusov et al., 2000; Altschul et al., 2005), and protein motif identification used CDD v3.18 (Marchler-Bauer et al., 2017) and Pfam v32.0 (Finn et al., 2016). Schematic representation of the Me. baoshan with main features was generated using DNA plotter v17.0.1 (Carver et al., 2009).

### Phylogenetic Analysis

The amino acid sequences of viruses in the family *Mimividae* including APMV (GeneBank accession number: HQ336222.2), Acanthamoeba castellanii mamavirus strain Hal-V (ACMV, JF801956.1), Samba virus (Mi. Samba, KF959826.1), Mimivirus shirakomae (Mi. shirakomae, AP017645.1), Mimivirus kasaii (Mi. kasaii, AP017644.1), Acanthamoeba polyphaga moumouvirus (Mo. mou, NC_020104.1), Moumouvirus australiensis (Mo. australiensis, MG807320.1), Saudi moumouvirus (Mo. saudi, KY110734.1), Megavirus chiliensis (Me. chiliensis, JN258408.1), Megavirus lba (Me. LBA, JX975216.1), Megavirus courdo11 (Me. courdo11, JX885207.1), Powai lake megavirus (Me. powailake, KU877344.1), Megavirus daqing (Me. daqing, MT663335.1), Tupanvirus deep ocean (Tp. deepocean, KY523104.1), and Tupanvirus soda lake (Tp. sodalake, MF405918.1), were retrieved from GenBank. Yasminevirus sp. GU-2018 strain A1 (Yasminevirus, UPSH01000001.1) was used as outgroup.

Orthologue sequences were extracted from the results of OrthoFinder v2.5.2 (Emms and Kelly, 2019), using MAFTT for multi-sequence alignment (msa) and RAxML for tree inference (Stamatakis, 2014), and five NCLDV core genes (Yutin et al., 2009), including major capsid protein, family B DNA polymerase, D5-like primase-helicase, transcription factor S-II, and virion packaging ATPase were chosen for building of the phylogenic trees.

In MEGA X v10.1.8 (Kumar et al., 2018), orthologue sequences were aligned using MUSCLE (Edgar, 2004) with default option in ALIGN program. The best evolutionary models were calculated for each core gene with MODELS program to be LG models with GAMMA distributed rates of 5 in all cases. Phylogenetic trees were generated using the maximum-likelihood method with LG + G model and 1,000 bootstrap replicates in PHYLOGENY program. Phylogenetic trees were visualized using Evolview v3 (Subramanian et al., 2019). Five core genes were concatenated using Seqkit v2.1.0 (Shen et al., 2016) and the concatenated tree was constructed similarly.

### Co-linearity Analysis

The synteny analysis between different mimivirus genomes was performed using MUMMER v4.00 (Marçais et al., 2018), and the average nucleotide identity (ANI) was calculated by JSpeciesWS v3.8.5 (Richter et al., 2016) based on BLAST+.

### Estimation of the Selection Pressure

Orthologous protein searches between Me. baoshan and Me. powailake were performed using BLASTP with an *e*-value threshold of 10^–5^ and maximum number of target sequence of 1. The pairwise alignments of orthologous proteins were converted into codon alignments using ParaAT v1.0 (Zhang et al., 2012). The rate of non-synonymous (dN) and synonymous (dS) substitutions and their ratio (dN/dS) were computed by PAML_X v1.3.1 using YN00 program (Yang, 1997; Xu and Yang, 2013) with default options.

## Results

### Genomic Sequencing, Assembly, and Annotation

The draft map genome of the Me. baoshan was constructed based on 3,443,297 reads of Illumina sequencing data. It was composed of 20 contigs, with a total of 1,197,945 bp in length, and had an average coverage of 828x (Tables 1, 2). To further improve the quality of genome assembly, Nanopore sequencing was carried out, and 43,349 Nanopore clean reads with mean length of 12,761 bp were assembled *de novo* into one contig of 1,224,839 bp in length. The sequence was improved by Illumina reads (Tables 1, 2), yielding the preliminary hybrid assembly. The sequences of terminal regions in the assembly were further analyzed using PCR and Sanger sequencing to correct errors (see below), and the final complete genome of Me. baoshan was obtained.

**TABLE 1:** Summary of clean DNA sequencing data.

Libraries	No. reads	Ave. size/bp	mapped reads/%	Ave. coverage
Nanopore	43,349	12,761	99.93	113
Illumina	3,443,297	152	98.13	828
				

**TABLE 2:** Statistics of genome assemblies.

Map	Draft	Complete
No. of contigs	20	1
Bases in all contigs (bp)	1,197,945	1,224,839
No. of large contigs (>1 kb)	13	1
Largest contigs length (bp)	603,082	1,224,839
N rate* (%)	0.001	0
GeneBank accession	MH046811.1-MH046830.1	MH046811.2

*N rate: percentage of uncertain nucleotide.

**FIGURE 1:**
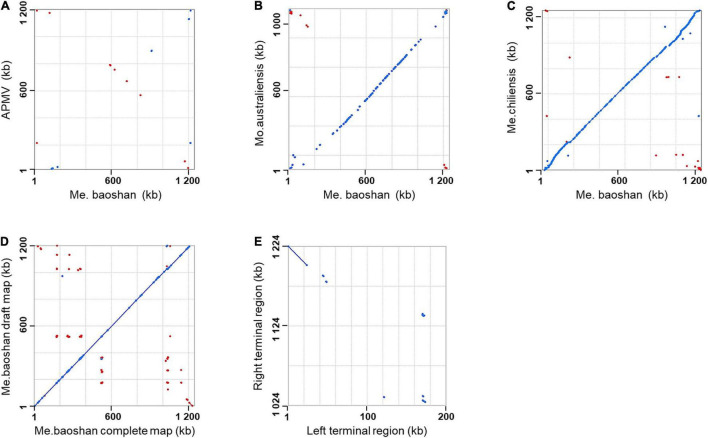
Synteny analysis of Me. baoshan complete genome with **(A)** APMV, **(B)** Mo. Australiensis, **(C)** Me. chiliensis, and **(D)** draft map of Me. baoshan. **(E)** Terminal region comparison between left and right terminal region of Me. baoshan genome. Each dot symbolized the best Mummer match between the regions of the two viruses in the same orientation (blue) or in reverse orientation (red).

Genome co-linearity analysis showed that Me. baoshan had a large quasi-perfect co-linearity in the center of the genome with Mo. australiensis and Me. chiliensis but had poor synteny with APMV overall (Figures 1A–C).

**FIGURE 2:**
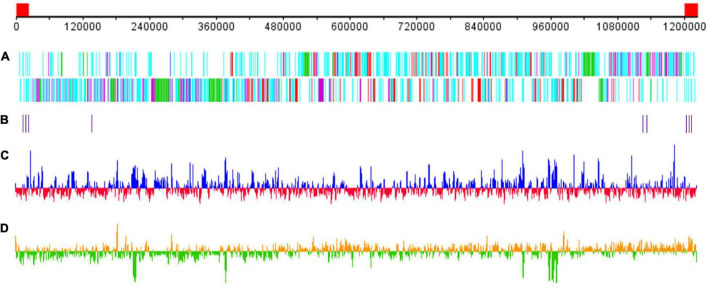
Schematic representation of the Me. baoshan genome. **(A)** Distribution of ORFs. Upper line and lower line represented plus and negative strand, respectively. Genes were color-coded according to the characteristics of their gene products. Green: FNIP repeat domain-containing proteins; purple: Ankyrin repeat domain-containing proteins; red: single copy proteins; blue: translation-related proteins; cyan: other proteins. **(B)** Distribution of tRNA genes. **(C)** GC content with higher or lower GC% compared with whole genome average in blue or red, respectively. **(D)** GC skew for 1000-bp window and 500-bp step with skew+ in orange and skew– in green. The red block in the genome scale line (top) represent the ITRs.

The GC content of Me. baoshan was 25.05%, similar with other megaviruses. A total of 1,062 open reading frames (ORFs) ranging from 30 to 2,909 amino acids (AA), with an average length of 338, were predicted. Nine tRNAs and 15 translation-related genes, including aminoacyl tRNA synthetase, eukaryotic translation initiation factor and peptide chain release factor, with homologs in other Megavirus, were also predicted (Figure 2; Arslan et al., 2011; Assis et al., 2017). The average distance between consecutive ORFs was 119-nt, resulting in a coding density of 88.36%. Five strict orphan open reading frames (ORFans), the nucleotide sequences of which were absent in other mimiviruses, were identified, with their lengths ranging from 29 to 57 AA.

### Comparative Genomics

**FIGURE 3:**
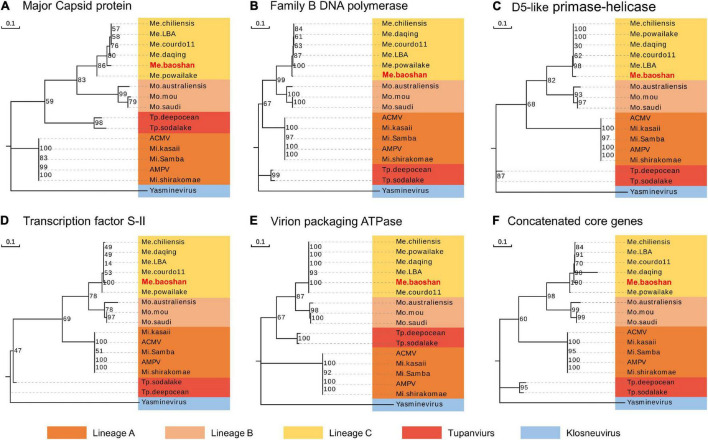
Molecular phylogenetic analysis based on the amino acid sequences of the NCLDV core genes, namely, **(A)** major capsid protein, **(B)** family B DNA polymerase, **(C)** D5-like primase-helicase, **(D)** transcription factor S-II, **(E)** virion packaging ATPase, and **(F)** the concatenated NCLDV core genes consisting of above five genes. Bootstrap consensus tree for 1000 replicates was computed using neighbor joining of maximum likelihood distances. Numbers on each branch represent percentage of trees containing that branch. Background colors represented different lineages of viruses.

To estimate the phylogenetic position of Me. baoshan in the family *Mimiviridae*, molecular phylogenetic analysis was performed based on NCLDV core genes, including major capsid protein, family B DNA polymerase, D5-like primase-helicase, transcription factor S-II, and virion packaging ATPase (Assis et al., 2017; Abrahão et al., 2018; Takahashi et al., 2021). Me. baoshan grouped with viruses of lineage C, close to Me. powailake, in all five phylogenetic trees based on individual core genes (Figures 3A–E), as well as the concatenated tree of five genes (Figure 3F), indicating that Me. baoshan belongs to lineage C of *Mimiviridae*.

Additionally, the average nucleotide identity (ANI) analysis of whole genome were performed to compare the nucleotide sequence divergence of Megaviruses. The ANIs of Me. baoshan and other Megavirus ranged from 86.54 to 88.56% with the alignment percentage ranged from 58.90 to 59.07% (Table 3). The result suggested that Me. baoshan was relatively distant from other isolates in the lineage C.

**TABLE 3:** Whole genome average nucleotide identities (ANI) between megaviruses.

ANI [% aligned]	Me. baoshan	Me. Daqing	Me. chiliensis	Me. courdo11	Me. powailake	Me. LBA
Me. baoshan	-	88.29 [58.57]	88.43 [59.63]	88.36 [59.40]	86.54 [58.36]	88.23 [59.14]
Me. daqing	88.56 [58.90]	-	93.84 [70.75]	93.96 [70.05]	89.68 [67.24]	93.83 [70.48]
Me. chiliensis	88.12 [59.86]	93.81 [68.99]	-	97.17 [72.51]	89.67 [66.69]	97.88 [73.35]
Me. courdo11	88.22 [59.67]	93.95 [69.72]	97.23 [73.28]	-	89.72 [67.71]	97.19 [72.41]
Me. powailake	86.68 [59.07]	89.80 [67.95]	90.07 [68.79]	90.03 [68.63]	-	89.92 [68.32]
Me. LBA	88.07 [60.88]	93.89 [70.58]	98.11 [75.79]	97.21 [73.83]	89.68 [68.99]	-

What’s more, 973 (91%) of the total 1,062 ORFs of Me. baoshan had homologs in Me. powailake, with an average amino acid residue identity of 80.85%. All these homologs were under purifying selection, as the average value of omega (dN/dS ratio, which was the ratio of non-synonymous mutations to synonymous mutations) was 0.100 ± 0.089 (median = 0.079), indicating that frequent synonymous mutations contributed to the nucleotide diversity of Megavirus.

All these results revealed that Me. baoshan was a new member of Mimivirus lineage C.

### Identification of Inverted Terminal Repeat Regions

The length of the draft map of Me. baoshan was 26 kb shorter than that of the complete one (Table 2), which raised a question whether one of terminal regions was missing in the draft map. This was confirmed when the draft map was compared with the complete map using co-linearity analysis, in which the two maps had high co-linearity all over the genome, except that the complete map had some extra sequences at the right end (Figure 1D). More interestingly, the extra sequences at the right end of the complete genome seemed to be homologous to the left end of the draft map, but in a reversed orientation (Figure 1D, bottom-right corner). When the sequences of the two extremities of the complete map of Me. baoshan were compared for co-linearity, a prefect synteny was seen (Figure 1E). Further sequence comparison of the two extremities confirmed the presence of 23-kb-long inverse repeats at both extremities.

**FIGURE 4:**
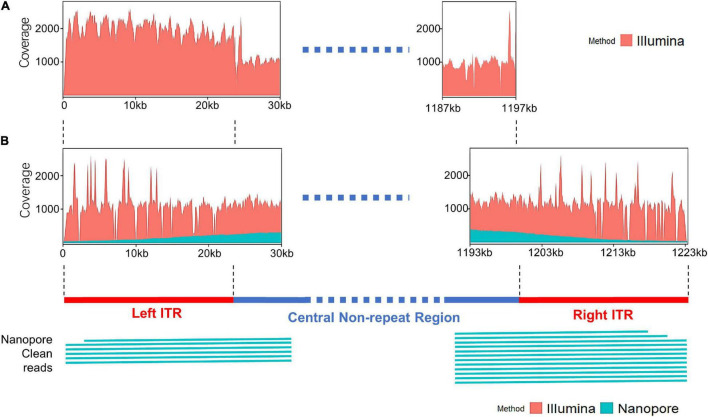
**(A)** The coverage plots of Me. baoshan draft genome by mapping Illumina reads against the draft map genome. **(B)** The coverage plots of Me. baoshan complete genome by mapping Illumina and Nanopore reads against the complete genome map, followed by schematic representation of Nanopore long reads (>26 kb) that mapped against the terminal regions of the complete map.

The distribution of sequencing reads at the terminal regions of linear genome was analyzed. When the Illumina reads were mapped against the draft map of Me. basoshan, the average coverage of the 23-kb terminal regions in the left end (1,973x) was twice that of the other region (828x) (Figure 4A), implying that there were two copies of the terminal sequences in the genome, and the Illumina reads of the right terminal region were mistakenly assembled as the left one, resulting in the absence of the right terminal region. Meanwhile, when Illumina reads were remapped against the complete map, the average coverage by Illumina reads of the left end region (1,046x) became similar to that of the right end region (1,004x) (Figure 4B).

### Inverted Terminal Repeat Sequence Verification

To further confirm the presence of ITR, reads of Illumina and Nanopore sequencing were analyzed in detail. Six and twelve Nanopore reads longer than 26 kb were found to cover the left and right ITR region, respectively, extending from the central non-ITR region toward the terminal of the genome, with average 90.13% nucleotide identity (Figure 4B). When the border areas between the ITRs and central non-ITR regions (left border area: 23,869–23,969 and right border area: 1,200,871–1,200,971) were examined, 289 and 208 Nanopore reads, as well as 1018 and 746 Illumina reads, were found to cover the left or right border areas, respectively.

**FIGURE 5:**
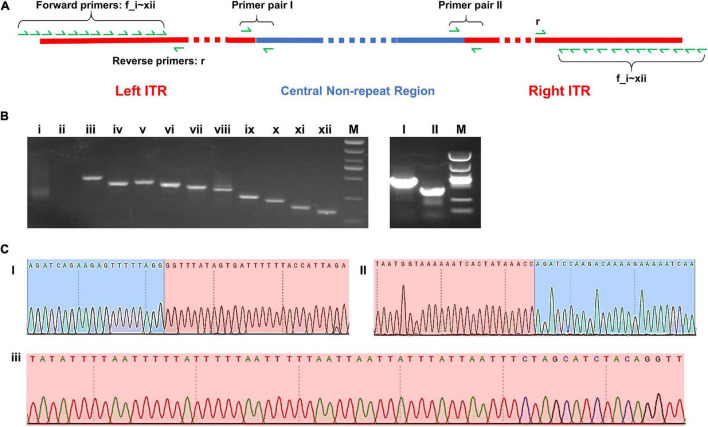
**(A)** Schematic diagram of the complete map of Me. baoshan genome with the location of PCR primers. **(B)** Agarose gel electrophoresis analysis of PCR product. Lanes i–xii: PCR products using 12 forward primers closing to the extremities of ITR region pairing with one reserve primers; Lanes I and II: PCR products over the junction of ITR and non-ITR region using primer pairs I and II, respectively. **(C)** Partial results of Sanger sequencing from lane I, II, and iii. Red background represented ITR region and blue background represented non-ITR region.

Two pairs of PCR primer covering the two border areas between ITRs and central non-repeat regions were designed, and the sequencing results of the PCR products were found to be consistent with the sequence of two border regions in the complete map (Figures 5C I,II).

In the preliminary hybrid assembly, some extra non-repeat sequences were presented in front of the left ITR and behind the right ITR. Standard PCR amplification and Sanger sequencing were performed to analyze these sequences (Figure 5). Twelve forward primers close to the extremities of ITRs (primers f_i, f_ii, … f_xii) and one reverse primer (primer r) were designed. Only primers within the ITRs yielded PCR products, whereas primers beyond ITRs yielded no PCR product (Figure 5B i,ii). It was concluded that the extended non-repeat sequences outside the ITRs were artifacts. The results of Sanger sequencing confirmed the terminal AT-rich nucleotide sequences at the ends of the genome (Figure 5C iii).

Seventy-five single-nucleotide variants (SNVs) were found between the left and the right ITR in the preliminary hybrid assembly, among which 47 SNVs brought in frame shift mutations in ORFs. Since the coverages of these regions with SNVs by Illumina sequencing were extremely low (ranged from 1x to 19x) compared with that of the whole ITRs (Figure 4B), PCR and Sanger sequencing were carried out to re-examine these SNVs in detail. Twelve PCR products ranging from 161 bp to 408 bp in length, the same as expected, were obtained and cloned, and 5–10 recombinants for each PCR product were subjected to Sanger sequencing. No variation was found among clones of each PCR product, suggesting that these SNVs were not real, and the linear genome of Me. baoshan contained two identical ITRs at both ends. The results of Sanger sequencing were remapped onto the preliminary hybrid assembly to correct the sequencing errors.

### Inverted Terminal Repeat Sequence Characterization

**FIGURE 6:**
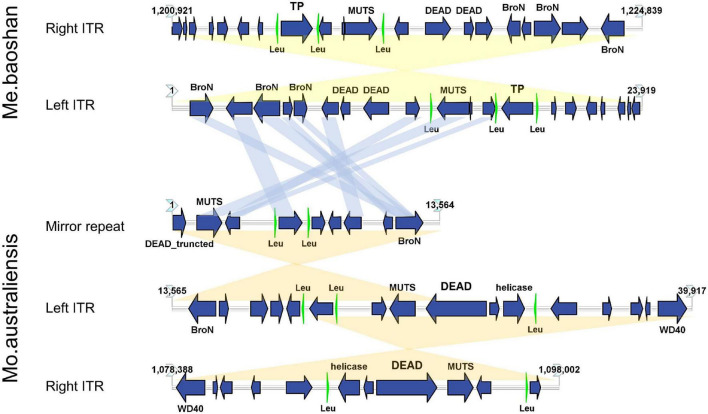
Map of ITRs of Me. baoshan and Mo. australiensis displaying the relative size and direction of ORFs (blue) and tRNA (green). Links in light blue represented the homologue proteins between different viral ITRs, and links in light yellow represented ITRs and mirror repeat within a viral genome. Map of Mo. Australiensis was made using sequence data in GeneBank (MG807320.1). BroN, Bro-N domain-containing protein; DEAD, DEAD-like helicase; MUTS, MUTS family DNA-binding domain protein; TP, transposase; WD40, WD40 repeat protein; Leu, tRNA-Leu.

The GC content of ITR was 23.54%, lower than that of the whole genome (25.05%). Each ITR encodes 20 ORFs, ranging from 37 to 539 AA, with an average length of 243 AA. Three tRNAs (Leu) were also predicted in each ITR (Figure 6). Three Bro-N domain-containing proteins, two DEAD-like helicases, one MUTS family DNA-binding protein, one ankyrin repeat domain-containing protein, and one transposase was annotated based on the conserved domain information, suggesting that the major function of proteins encoded in ITRs were related to DNA replication and repair.

## Discussion

Next-generation sequencing technologies have provided powerful tools for genomic studies. However, technology has its limitation. Illumina sequencing has relatively short reads that may not be suitable to analyze genomes with high content of long repeats. Nanopore sequencing has much longer reads, whereas its accuracy is not high enough. Hybrid sequencing by combining different technologies may provide a more comprehensive view of the genome (Gavrielatos et al., 2021; Lin et al., 2021). In the current study, Nanopore and Illumina sequencing technologies were used to analyze the genome of Me. baoshan, and the results showed that it was a new member of Mimivirus lineage C. What’s more, a pair of 23 kb-long ITRs was uncovered at both extremities of the linear genome, one of which was missing when only Illumina sequencing data was used for genome assembly.

Many NCLDVs, like poxvirus, ASFV, and chlorovirus, have ITRs ranged from 2.6 kb to 22 kb in length (Strasser et al., 1991; Meireles and Costa, 1994; Tulman et al., 2004, 2006; Mitsuhashi et al., 2014; Quispe et al., 2017). APMV is known to contain short inverted repeats of 900 bp near two extremities (Raoult et al., 2004). Me. baoshan is the first megavirus found to have long ITRs, and its 23-kb ITRs, to our knowledge, is the longest ITR reported in virus.

Among 32 genomic assemblies of *Mimiviridae* in NCBI NR database, only two mimiviruses, Mo. australiensis (MG807320.1, lineage B mimivirus) and Cotonvirus japonicus (AP024483.1, proposed new lineage mimivirus), were based on third-generation sequencing technology. Using the assembly data in the database, we identified two 19-kb-long ITRs at both extremities in Mo. australiensis genome, with nine SNVs between two ITRs. Similarly, two 5-kb-long ITRs were identified in C. japonicas. However, in the genome of Mo. australiensis, there was a mirror repeat at left ITR region, which was absent at right ITR (Figure 6). No such mirror repeat was found in Me. baoshan. Behind the right ITR of C. japonicas, there was extra 32-kb-long non-repeat region. No other mimiviruses were found to contain ITRs. Since most genome assemblies were based on Illuminia technology, and one of the two ITRs was missed in our Illumina-based draft map of Me. baoshan, it is worth to further examine the terminal sequences of these mimiviruses.

In addition, there were several amoeba giant viruses whose linear genome assembly was based on PacBio sequencing, including BsV (Klosneuvirus) (Deeg et al., 2018) and Pandoravirus celtis (Pandoravirus) (Legendre et al., 2019). No similar long ITRs were seen in their genome assemblies. More detailed studies are needed to resolve the terminal structure of linear viral genome of amoeba giant viruses.

Unlike poxvirus and some other viruses, the ITR of which were relatively conserved among members of the same taxonomic group (Tulman et al., 2004, 2006; Mitsuhashi et al., 2014), the mimiviral long ITR was not highly conserved among viruses. No long nucleotide sequences were found to be significantly homologous to Me. baoshan ITR in database. Although, nine of 20 ORFs (45%) encoded by the ITR of Me. baoshan had homologs in that of Mo. australiensis, their homologies in amino acid sequences were not very high (29.92% identity with 66% coverage to 87.74% identity with 100% coverage).

Inverted terminal repeat has been found to be important for the stability and replication of linear DNA replicons and virus genomes (Blackburn, 1991). The approximately 900 bp inverted repeats found in APMV was suggested to play an important role in virus genome replication (Raoult et al., 2004; Yoshida et al., 2011; Chelikani et al., 2014; Akashi and Takemura, 2019). Apart from that, since long ITRs found in Me. baoshan and some other mimiviruses encodes many genes, two copies of ITR in the genome might mean more efficient expression of these genes (Earley et al., 2020). This is supported by our observations that many of the genes in ITR were highly expressed in the immediate early stage after infection (unpublished data). The significance of this phenomenon is worth further investigation.

## Data Availability Statement

The datasets presented in this study can be found in online repositories. The names of the repository/repositories and accession number(s) can be found in the article/supplementary material.

## Author Contributions

YX and JZ contributed to conception and design of the study and wrote sections of the manuscript. YX and HC conducted the experiments. YX performed the statistical analysis and wrote the first draft of the manuscript. All authors contributed to manuscript revision and read and approved the submitted version.

## Conflict of Interest

The authors declare that the research was conducted in the absence of any commercial or financial relationships that could be construed as a potential conflict of interest.

## Publisher’s Note

All claims expressed in this article are solely those of the authors and do not necessarily represent those of their affiliated organizations, or those of the publisher, the editors and the reviewers. Any product that may be evaluated in this article, or claim that may be made by its manufacturer, is not guaranteed or endorsed by the publisher.

## Footnotes

1
www.snapgene.com
